# Tolerance to heated egg in egg allergy: Explanations and implications for prevention and treatment

**DOI:** 10.1002/clt2.12312

**Published:** 2023-12-21

**Authors:** Audrey Leau, Sandra Denery‐Papini, Marie Bodinier, Wieneke Dijk

**Affiliations:** ^1^ INRAE UR BIA Nantes France

**Keywords:** baking, egg allergy, heating, oral immunotherapy, primary prevention

## Abstract

Hen's egg allergy is the second most frequent food allergy found in children. Allergic symptoms can be caused by raw or heated egg, but a majority of egg‐allergic children can tolerate hard‐boiled or baked egg. Understanding the reasons for the tolerance towards heated egg provides clues about the molecular mechanisms involved in egg allergy, and the differential allergenicity of heated and baked egg might be exploited to prevent or treat egg allergy. In this review, we therefore discuss (i) why some patients are able to tolerate heated egg; by highlighting the structural changes of egg white (EW) proteins upon heating and their impact on immunoreactivity, as well as patient characteristics, and (ii) to what extent heated or baked EW might be useful for primary prevention strategies or oral immunotherapy. We describe that the level of immunoreactivity towards EW helps to discriminate patients tolerant or reactive to heated or baked egg. Furthermore, the use of heated or baked egg seems effective in primary prevention strategies and might limit adverse reactions. Oral immunotherapy is a promising treatment strategy, but it can sometimes cause significant adverse events. The use of heated or baked egg might limit these, but current literature is insufficient to conclude about its efficacy.

## 1 INTRODUCTION

Food allergy is a pathological reaction of the immune system triggered by the ingestion of a food antigen. Exposure to very small number of allergenic foods can trigger clinical symptoms, such as gastrointestinal disorders, urticaria and airway inflammation that range in severity from mild to life‐threatening.^1^ Reactions can rarely be fatal and are caused by anaphylactic shock. Food allergy prevalence and severity seem to be increasing, and a recent analysis of European food allergy prevalence found a life‐time overall prevalence of self‐reported physician‐diagnosed food allergy of 6.6%.^2^ Among the risk factors identified for food allergies are genetics, including a family history of allergy, having parents born in East Asia and the presence of a filaggrin gene mutation.^3^ Beyond genetic factors, environmental factors such as microbial exposure, food introduction and serum vitamin D levels modulate food allergy risk, and are likely key to the recent rise in food allergy prevalence and severity.^3^

Among food allergies, hen's egg allergy is the second most frequent food allergy found in young children (∼2.7% life‐time self‐reported physician‐diagnosed, in Europe).^4^ Most egg allergies develop in the first year of life and are frequently outgrown during childhood or adolescence.^5^ The most common symptoms of hen's egg allergy in children are IgE‐mediated reactions, such as erythema, urticaria, eczematous rash, abdominal pain, diarrhoea and vomiting.^6^ The current treatment for egg allergy involves strict dietary avoidance or minimised contact with the allergen. As an alternative to an avoidance diet, oral immunotherapy (OIT) has been investigated. OIT involves the ingestion of small doses of egg protein by an allergic individual. This dose is gradually increased over time to improve tolerance and further desensitize the allergic patient. Beyond treatment strategies, primary prevention strategies are actively studied to prevent the development of egg allergy. These prevention strategies notably involve the early introduction of specific forms of egg in young infants.

**FIGURE 1:**
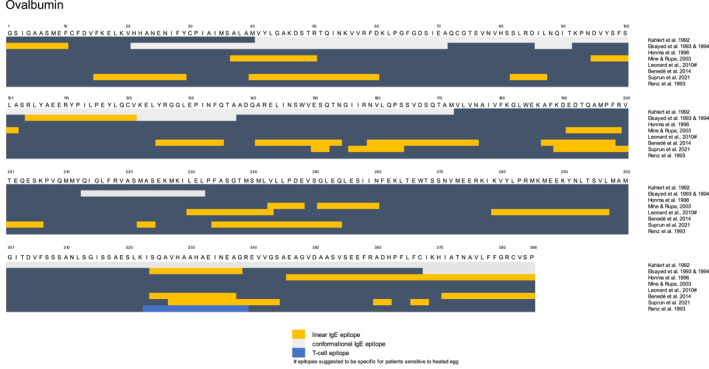
Overview of published epitopes for Ovalbumin (Gal d 2, OVA).^23^, ^24^, ^25^, ^26^, ^27^, ^28^, ^29^, ^30^, ^31^

**FIGURE 2:**
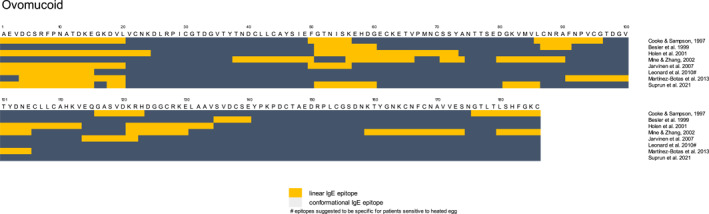
Overview of published epitopes for Ovomucoid (Gal d 1, OVM).^25^, ^28^, ^29^, ^32^, ^33^, ^34^, ^35^, ^36^

The main allergens of hen's eggs are found in the egg white (EW), which consists predominantly of water and 11% proteins of over 40 different types.^7^ The most abundant EW proteins have been identified as allergens: ovomucoid (OVM) (Gal d 1, approximately 11%), ovalbumin (OVA) (Gal d 2, approximately 54%), ovotransferrin (Gal d 3, approximately 12%) and lysozyme (Gal d 4, approximately 3.5%) (see Table 1).^6^, ^7^ Two allergens have also been identified in egg yolk (serum albumin – Gal d 5, YGP42—Gal d 6), but their clinical significance remains to be further established.^21^, ^22^ OVM and OVA are the immunodominant allergens based on specific serum IgE (sIgE) levels. Clinical reactivity occurs towards specific amino acid sequences of proteins, which are called epitopes. Linear epitopes are defined as continuous sequences of amino acids capable of binding IgE, whereas conformational epitopes are formed by amino acids that are spatially close in the protein 3D conformation but distant in the protein sequence. Known linear and conformational epitopes of OVA and OVM are noted in Figures 1 and 2.^37^

**TABLE 1:** Major allergens in hen's eggs.

Allergen	Name	Localization	MW (kDa)	Heat stability	Digestion stability	References
Gal d 1	Ovomucoid	EW	28	**Yes**	**Moderate**, pepsin‐sensitive but IgE epitopes remain after digestion	8, 9, 10, 11
Gal d 2	Ovalbumin	EW	44	**No**	**Yes** in native form **No** upon heating	9, 10, 11, 12, 13
Gal d 3	Ovotransferrin	EW	78	**No**	**No**	14, 15
Gal d 4	Lysozyme	EW	14	**Moderate**	**Yes**, but possible precipitation upon gastro‐intestinal digestion	15, 16, 17, 18, 19
Gal d 5	Serum albumin	Egg yolk	69	**No**	**No**	18
Gal d 6	YGP42	Egg yolk	35	**Yes**	**No**	20

Abbreviation: EW, egg white.

Allergic symptoms can be caused by the consumption of raw or heated eggs. Nonetheless, a majority of egg‐allergic children (between 63% and 83%) can tolerate hard‐boiled or baked (>100°C) egg.^38^, ^39^, ^40^ Two phenotypes of egg‐allergic children can thus be distinguished; patients reactive only to raw egg and patients reactive to all forms of egg. Understanding the reasons for the tolerance of heated egg by some but not all egg‐allergic patients might provide clues about the molecular mechanisms involved in hen's egg allergy sensitization and allergic reactions in general. Furthermore, the differential allergenicity of the different forms of eggs might be exploited to prevent or treat egg allergy. In this review, we aim to discuss in detail (i) why some patients are able to tolerate heated egg; by discussing the structural changes of EW proteins upon heating and their impact on EW immunoreactivity, as well as patient characteristics, and (ii) to what extent heated egg (white) might be useful for primary prevention strategies or oral immunotherapy. As OVM and OVA are the immunodominant allergens, the impact of heating on these allergens will be discussed in detail.

## 2 WHY DO SOME PATIENTS TOLERATE HEATED EGG?

### 2.1 EW heating can modify immunoreactive epitopes and protein digestion

#### 2.1.1 Structural characteristics of heated EW

To understand why heated eggs are better tolerated by egg‐allergic patients, the physiochemical and structural changes occurring during the heating of EW (proteins) need to be considered first. Various types of heat treatment can be applied to egg or EW to make different food products, including egg (white) pasteurization (58–65.5°C for 2.5–5 min for liquid egg, 55–57.2°C for liquid EW),^41^ boiling (100°C for 5–30 min), scrambling (pan‐cooked, 4–6 min), and baking. Baked egg is characterized by a method of cooking that uses prolonged dry heat, normally in an oven, in the absence (e.g. oven‐baked egg) or the presence of wheat proteins (e.g. muffins or biscuits). With increasing temperature, EW proteins progressively unfold and denature, which results in protein aggregation and coagulation, giving heated EW its milky white colour.^42^ Beyond aggregation, heating can also induce the so‐called Maillard glycation, which is a complex set of chemical reactions in which free amino groups of proteins interact with the carbonyls of reducing sugars.^43^ Maillard glycation takes place naturally in the presence of sugars but is accelerated by heat and is frequently observed during baking and cooking as food browning.^43^ The progressive unfolding of egg proteins and their glycation upon heating depends on (i) the time and temperature of heating, (ii) the characteristics of the EW proteins, and (iii) environmental factors (e.g. pH, ionic strength, the presence of other protein sources such as wheat gluten).^43^

EW aggregation generally starts at 60°C—the denaturation temperature of ovotransferrin—and further accelerates at 70°C—the denaturation temperature of OVA.^6^, ^44^ OVA contains 6 cysteine residues (Cys12, Cys31, Cys74, Cys121, Cys368, and Cys383) of which Cys74 and Cys121 form a disulphide bond in the native state.^45^ Following heat denaturation, a hydrophobic C‐terminal region containing a sulfhydryl group (Cys368) is exposed to the surface and contributes to OVA aggregate formation.^46^, ^47^ OVA aggregation is rapid and results in the formation of thin strands (linear aggregates) or denser particles depending on the physicochemical conditions used during heating (pH, ionic strength, protein concentration).^12^, ^14^, ^48^ In contrast to the heat‐labile nature of OVA, OVM is highly resistant to heat thanks to its conformation of three tandem domains with intra‐ but not inter‐domain disulphide bonds.^8^, ^44^, ^49^ Only prolonged heating at temperatures above 90°C (e.g. boiling >30 min) results in the formation of an irreversible denatured state, indicating that OVM will remain in a natural state in more transiently heated forms of egg.^8^, ^50^ One particular situation in which OVM does aggregate is when OVM is heated in the presence of wheat. Indeed, OVM solubility is markedly decreased when EW is mixed with wheat gluten and heated, due to the formation of high‐molecular weight complexes with gluten.^51^, ^52^, ^53^ For this reason, egg baked in the presence of wheat should be clearly distinguished from other forms of heated egg in scientific studies.

#### 2.1.2 Heating can modify immunoreactive epitopes

The heat‐induced changes in EW proteins impact allergen epitopes. Allergens have two types of epitopes; T‐lymphocyte epitopes that are recognized by T‐lymphocytes following protein processing and presentation by antigen‐presenting cells, and B‐cell or IgE epitopes. These IgE epitopes are protein regions capable of binding and cross‐linking IgE, produced by plasma cells from memory B‐cells. Cross‐linking of the IgE‐FcεRI complex on the surface of mastocytes or basophils by an allergen causes their degranulation and the release of mediators (e.g. histamine) that provoke allergic symptoms. As IgE epitopes can either be linear or conformational, heat treatment might destroy conformational epitopes or mask linear epitopes due to protein aggregation or glycation, which might change epitope accessibility or alternatively generate new epitopes.^54^

The immunoreactive epitopes in OVA have been identified as a combination of conformational and linear epitopes.^23^ Conformational epitopes have been localized to the regions aa41‐172 (located at the surface of OVA) and aa367‐385 ^23^, ^24^ (Figure 1). Due to the heat lability of OVA, these conformational epitopes are likely lost upon heating. Different linear epitopes have also been identified, although with significant disparity between studies.^24^, ^25^, ^26^, ^27^, ^28^, ^29^ One linear epitope located around aa367‐385 was highlighted by multiple studies and was recognized by 80% of sera from egg‐allergic patients in one study, underlining its immunological importance.^23^, ^24^, ^25^, ^27^ It is of interest to note that this particular epitope is also in a region that aggregates and is glycated upon heating, suggesting that this epitope might become partially masked.^46^, ^47^ Other aggregation ‘hotspots’ for OVA have been identified at Cys31 and Cys121, which are both in proximity to linear OVA IgE epitopes (aa16‐30 and aa125‐134).^25^, ^29^, ^47^ ‘Hotspots’ of glycation were found at Lys190 (within the epitope aa 189–199) for dry heated samples and at Lys123 (near epitope aa 125–134) for wet heated samples.^25^, ^29^, ^47^, ^55^ These structural changes of OVA epitopes induced by heating and/or glycation lower the recognition of OVA epitopes by sIgE of patients, as assessed using Western blot and/or ELISA (see Table 2). Some linear epitopes do persist, as heat treatment of OVA does not fully abolish sIgE reactivity of patient sera^9^ (see Table 2). Indeed, two linear epitopes (aa 229–243, 280–297) were suggested to be specific for patients sensitive to extensively heated egg.^28^

**TABLE 2:** IgE patient serum reactivity against native and heated EW proteins.

Protein fraction	Heating	Heating conditions	Product	Western blot/dot blot	ELISA	References
Egg patient serum IgE reactivity						
EW or whole egg	4–6 min	Natural, EW	Scrambled EW	=	N/A	38, 56
	8–10 min 90°C	Natural, EW	Boiled EW	↓	N/A	
	30 min 176°C	Whole egg (1/3) + wheat matrix/muffin	Muffin	↓↓	N/A	
	3 min 260°C	Whole egg (1/3) + wheat matrix/muffin	Waffle	↓↓	N/A	
EW	10 min 100°C	Liquid (natural)		=	= OVM & OVA	57
	30 min 100°C			↓	= OVM, ↓↓ OVA	
	20 min 170°C			↓	= OVM, ↓ OVA	
	Fried			↑	= OVM, ↓ OVA	
OVA	15 min 95°C	Liquid (pH 7)		N/A	↓	15
OVA	15 min 90°C	Liquid (pH 7)		N/A	↓	10
	96 h 50°C	Dry with glucose		N/A	↓	
OVA	30 min 100°C	Liquid		↓	N/A	9
OVA	30 min 100°C	Liquid (pH 9.6)		N/A	=/↓	58
	60 min 100°C	Liquid (pH 9.6)		N/A	↓	
	30 min 100°C	Liquid (pH 9.6) with glucose		N/A	=/↓	
	60 min 100°C	Liquid (pH 9.6) with glucose		N/A	↓	
OVA	6 h 80°C	Liquid (pH 9)		N/A	↓↓	13
	6 h 80°C	Liquid (pH 5)		N/A	↓	
OVA	3 h 65°C	Dry		N/A	=	59
	3 h 65°C	Dry with mannose		N/A	↓	
OVA	6 h 50°C	Dry with glucose		N/A	↓	55
OVA	10 days 37°C	Dry		N/A	=	60
	10 days 37°C	Dry with D‐glucose		N/A	=/↓	
	10 days 37°C	Dry with D‐mannose		N/A	=/↓	
	10 days 37°C	Dry with D‐allose		N/A	↓↓	
	10 days 37°C	Dry with D‐galactose		N/A	↓↓	
	10 days 37°C	Dry with L‐idose		N/A	↓	
OVA	30 min at 65°C	Liquid		=	=	61
	30 min at 65°C	Liquid with methylglyoxal		↓	↓	
	30 min at 65°C	Liquid with glyoxal		↓	↓	
	30 min at 65°C	Liquid with butanedione		=	=	
	15 min at 95°C	Liquid		↓	↓	
	15 min at 95°C	Liquid with methylglyoxal		↓	↓	
	15 min at 95°C	Liquid with glyoxal		↓↓	↓	
	15 min at 95°C	Liquid with butanedione		↓	↓	
OVA	6 h 80°C	Liquid (pH 9)		N/A	↓↓	47
	6 h 80°C	Liquid (pH 9) with glucose		N/A	↓↓	
	72 h 55°C	Dry		=	=	
	72 h 55°C	Dry with glucose		↓	↓	
OVM	45 min 100°C	Natural (whole egg)		N/A	↓	50
OVM	15 min 95°C	Liquid (pH 7)		N/A	↓	15
OVM	15 min 90°C	Liquid (pH 7)		N/A	↓	10
	96 h 50°C	Dry with glucose		N/A	↑	
OVM	30 min 100°C	Liquid		=	N/A	9
OVM	48 h 60°C	Dry with galactooligosaccharide		N/A	↓	62
	48 h 60°C	Dry with fructooligosaccharide		N/A	=	
	48 h 60°C	Dry with mannosan		N/A	=	

*Note*: Samples in grey are samples heated in the presence of sugars.

Abbreviations: EW, egg white; OVA, ovalbumin; OVM, ovomucoid.

For OVM, both linear and conformational epitopes play a role in OVM allergy and their relative importance likely differs per patient.^63^ Some OVM‐sensitized individuals might not recognize linear epitopes at all.^32^ Overall, OVM heating moderately reduces serum IgE binding, but most OVM‐reactive patients still react significantly to heated OVM (see Table 2). For some patients, IgE reactivity even increases upon glycation, suggesting the appearance of novel epitopes^9^, ^10^, ^64^ (see Table 2). As OVM does not aggregate and only irreversibly denatures upon prolonged heat exposure (boiling >30 min), it is likely that many OVM IgE‐binding epitopes remain accessible in moderately heated OVM and EW, although no detailed molecular studies on OVM epitopes and heating have been performed yet. Nonetheless, the reduced epitope accessibility of OVA and, to a lesser extent, OVM likely explains the reduced capacity of sIgE of egg‐allergic patients to bind to heated or baked EW (see Table 2). The length of heat treatment seemed to be the most determinant for the loss of EW IgE reactivity following heating, which is probably linked to the gradual chemical modification of linear IgE epitopes^56^, ^57^ (see Table 2).

#### 2.1.3 Heating alters egg protein digestion and absorption

Beyond the changes in conformational or linear epitopes, heating also impacts the digestibility of EW proteins and their absorption. For food allergens to trigger allergic symptoms, the allergen must conserve at least 2 epitopes following digestion and be absorbed in an immunologically active form across the epithelial barrier. An extensive study that used EW heated at different temperatures and times (56°C for 32 min; 65°C for 30 min; 100°C for 5 min) showed that heating significantly increased EW protein digestion.^16^ Gastric digestion was highest following heating at 65°C for 30 min, whereas gastro‐intestinal digestion was highest upon heating at 100°C for 5 min.^16^ The increased digestibility of EW following heating may at least be partially explained by the increased digestibility of OVA following heating.^9^, ^10^, ^11^, ^12^, ^13^ Indeed, whereas native OVA has a high resistance to gastric digestion, heat‐aggregated OVA is more easily digested and the peptides that are released are different.^12^, ^13^ The reactivity of basophils sensitized with sera from egg‐allergic patients was also significantly reduced but not abolished following the heating and digestion of OVA, compared to unheated, digested OVA.^9^, ^13^ In contrast to heating alone, the glycation of OVA lowers its digestibility and the peptides released are different than unheated or heated OVA.^10^, ^65^ It remains to be clarified to what extent heated or glycated OVA crosses the barrier in an immunologically active form. Two studies indicated that the heating of OVA significantly lowered the amount of circulating OVA following oral gavage in mice, while another study showed that heated OVA was unable to activate pre‐sensitized basophils following transport across the intestinal barrier.^9^, ^66^, ^67^

In contrast to OVA, OVM gastro‐intestinal digestion is not significantly affected by heating due to its high thermal stability.^9^, ^10^ OVM is digested by gastric and gastro‐intestinal fluids, but its digestion is not complete as epitopes recognized by IgE in human sera remain present.^8^, ^10^, ^11^ Using basophils sensitized with sera from egg‐allergic patients, gastro‐intestinal degradation but not heating of OVM significantly reduced basophil reactivity.^9^ Glycation of OVM also did not affect gastro‐intestinal digestion.^10^ Heating did lower OVM immunoreactivity following passage of the epithelial barrier compared with native OVM, but the underlying mechanisms remain to be clarified.^9^

**Summary:**

1

- OVA is more heat labile than OVM
- OVM only becomes heat labile in the presence of wheat or upon prolonged heating (>30 min)
- Heating impacts OVA conformational and some, but not all linear epitopes
- Heating has limited impact on conformational and linear OVM epitopes
- OVA sensitivity to gastro‐intestinal digestion is increased by heating, but reduced by glycation
- OVM is sensitive to gastro‐intestinal digestion, but this is not impacted by heating or glycation

### 2.2 Heating lowers egg allergic reactivity

#### 2.2.1 Heating egg lowers allergic sensitization capacity

To explain why certain patients tolerate heated or baked egg, multiple studies have studied how heating impacts the capacity of EW (proteins) to sensitize or provoke an allergic reaction. For allergic sensitization, data on the sensitization capacity of raw versus heated egg are only available for mice studies and are largely inconclusive. In one study, a significant reduction of total IgE and OVA sIgE levels was found when mice were sensitized to heated EW, compared to raw EW.^67^ In contrast, using a short heating time (10 min 80°C), another study found that mice sensitized with heated EW had significantly higher total IgE and OVA and OVM sIgE levels compared with raw EW^54^ (see Table 3). Studies using OVA to sensitize mice are more consistent and show that mice sensitized with heated OVA (10 min at 70°C or 6 h at 80°C) have modestly lower OVA sIgE compared to mice sensitized with native OVA^68^, ^69^ (see Table 3). Furthermore, IgG2a levels – indicative of a shift towards a Th1 helper profile in detriment of the Th2 response – were significantly higher in mice sensitized with heated OVA compared to native OVA.^68^, ^69^, ^70^ Interestingly, the sensitization capacity of heated OVA was found to be dependent on the aggregation process: small, linear aggregates of OVA formed at pH 9 (near natural pH of stored EW) and low ionic strength displayed a reduced allergic potential compared to large, spherical agglomerated aggregates formed at pH 5 and high ionic strength.^69^

**TABLE 3:** Allergy sensitization and allergic reactions against native and heated EW proteins in mice studies.

Protein fraction	Heating conditions	Protein conditions	Structure	sIgE levels (vs. Native)	IgG levels (vs. Native)	Mice	Sensitization	References
Allergic sensitization								
Mouse								
EW	5 min 100°C	Liquid	Aggregate	↓ (OVA)	N/A	Heterozygous OVA23–3	Oral *Powder in food, heated and native*	67
	15 min 80°C		Aggregate	↓ (OVA)	N/A			
	40 min 121°C		Aggregate	↓ (OVA)	N/A			
EW	10 min 80°C	Liquid	HMW aggregate	↑ (OVA, OVM)	↓ (OVA, OVM) IgG1	BALB/c	Oral *Gavage with heated and native OVA + CT*	54
OVA	10 min 70°C	N/S	Aggregate	↓	↑ IgG2a, ↑ IgG1	BALB/c	I.p.	68
OVA	6 h 80°C	pH 9, liquid	Small linear aggregates	↓	↑ IgG2a, = IgG1	BALB/cJ	I.p.	69, 70
	6 h 80°C	pH 5, liquid	Large aggregates	=	↑ IgG2a, = IgG1	BALB/cJ	I.p.	

Protein fraction	Heating conditions	Protein conditions	Structure	Symptoms (vs. Native)	Mice	Sensitization	Provocation	References
Allergic reaction								
Mouse								
EW	5 min 100°C	Liquid	Aggregate	↓ intestinal inflammation, diarrhoea	Heterozygous OVA23–3	Oral *Powder in food, heated and native*	N/A	67
	15 min 80°C		Aggregate	↓ intestinal inflammation, diarrhoea				
	40 min 121°C		Aggregate, glycated	↓ intestinal inflammation, diarrhoea				
EW	10 min 80°C	Liquid	HMW aggregate	Provocation with native := Clinical symptom score	BALB/c	Oral *Gavage with heated and native OVA + CT*	Oral and i.p *Heated and native OVA*	54
				Provocation with heated: ↓ clinical symptom score				
OVA	15 min 100°C	Liquid	Aggregate	↓ symptoms (maintenance of voluntary wheel running)	BALB/c	I.p. *Native OVA*	Oral gavage *Heated OVA*	66
OVA	10 min 70°C	N/S	Aggregate	↓ diarrhoea ↓ mMCP1	BALB/c	I.p. *Heated and native OVA*	Oral gavage *Heated and native OVA*	68
OVA	30 min 100°C	Liquid	N/A	↓ clinical symptoms (temperature)	C3H/HeJ	Oral *Gavage with native OVA + CT*	Oral gavage *Heated OVA*	9
OVA	6 h 80°C	pH 9, liquid	Small linear aggregates	↓ ear thickness, mMCP1	BALB/cJ	I.p. *Heated OVA, pH 9*	Oral gavage *Native OVA*	69, 70
	6 h 80°C	pH 5, liquid	Large aggregates	= Ear thickness, mMCP1	BALB/cJ	I.p. *Heated OVA, pH 5*	Oral gavage *Native OVA*	
OVA	2 or 7 days at 50°C at 65% humidity in the presence of glucose	Dry	Polymers	↓ IgE levels	dYY	I.p. *Native and glycated OVA*	N/A	71
OVA	6 weeks 50°C in the presence of glucose	Liquid	N/A	↑ IgE levels ↑ clinical symptoms (temperature)	BALB/c	I.p. *Native OVA and glycated OVA*	Oral gavage *Native OVA*	72
OVM	30 min 100°C	Liquid	N/A	↓ clinical symptoms (temperature)	C3H/HeJ	Oral *Gavage with native OVM + CT*	Oral gavage *Heated OVM*	9

Protein fraction	Heating	Heating conditions	Species	Basophil reactivity	References
Basophil reactivity					
Mouse					
OVA	10 min 70°C	N/S	Mouse	= Basophil mediator release	68
	10 min 95°C	N/S	Mouse	↓ basophil mediator release	
OVA	6 h 80°C	Liquid (pH 5)	Mouse	↓↓ basophil mediator release	70
OVM	30 min 100°C	Liquid	Mouse	= Basophil activation	9

*Note*: In grey: protein samples heated in the presence of sugars.

Abbreviations: CT, cholera toxin; EW, egg white; I.p., intraperitoneal; N/S, not specified; OVA, ovalbumin; OVM, ovomucoid.

Only few studies have investigated the impact of glycation on the sensitization capacity of OVA. Two studies showed a reduction in serum IgE levels following the sensitization of mice with glycated OVA compared with native OVA.^67^, ^71^ In contrast, a more recent study using heavily glycated OVA showed increased IgE levels and a stronger reduction in body temperature compared with intraperitoneal sensitization with native OVA.^72^ These opposing results are likely due to the extent of glycation and the heating temperature used to glycate OVA in the different studies and further studies are needed to clarify the impact of the extent of glycation on sensitization to OVA (Table 3). No data on allergic sensitization of heated and/or glycated OVM versus native OVM are currently available.

#### 2.2.2 Heating egg lowers egg allergic reactions

Numerous mice studies have investigated the capacity of heated EW (protein) to elicit allergic symptoms (see Table 3). In accordance with the observations in patients, all studies demonstrated a reduction in allergic symptoms when mice are sensitized and/or elicited with heated EW (protein)^9^, ^54^, ^66^, ^67^, ^69^, ^70^ (see Table 3). Pablos‐Tanarro and colleagues used an extensive cross‐over design in which mice were sensitized to native or heated EW and provoked with either native or heated EW.^54^ In this study, provocation with heated EW resulted in lower allergic symptoms in all mice compared to native EW, while the combined sensitization and provocation with heated EW resulted in the lowest overall clinical symptoms.^54^ In line with these studies, the reactivity of basophils sensitized with sera from egg‐allergic patients or sensitized mice was significantly reduced upon exposure to heated OVA or OVM, when compared to the native protein (see Tables 3 and 4). No studies have, to our knowledge, investigated the elicitation capacity of glycated OVA or OVM in mice or using basophils.

**TABLE 4:** IgE patient serum reactivity against native and heated EW proteins.

Protein fraction	Heating	Heating conditions	Species	Basophil reactivity	References
Basophil reactivity					
Human					
OVA	30 min 100°C	Liquid	Human	= Basophil activation ↓ After passage Caco‐2 barrier	9
OVA	6 h 80°C	Liquid (pH 9)	Human	↓↓ Basophil mediator release	13
	6 h 80°C	Liquid (pH 5)	Human	↓↓ Basophil mediator release	
OVM	30 min 100°C	Liquid	Human	= Basophil activation ↓ After passage Caco‐2 barrier	9

Protein fraction	Heating	Heating conditions	Product	SPT	Patient characteristics	References
Skin prick tests						
Whole egg	180°C for 20 min	Whole egg (1/6^th^) + wheat matrix	Muffin	↓ or = dependent on patient, compared to EW		74
EW, egg yolk, or whole egg	200°C for 25 min	EW, egg yolk, or whole egg baked in an oven		↓ compared to uncooked egg	36 children with egg allergy	73
EW, egg yolk, or whole egg	94°C for 18 min	Hardboiled egg, EW, or egg yolk		↓↓ for 43% (egg), 33% (EW), 72% (egg yolk) of children, = for the rest of children for egg and EW, ↓ for egg yolk	54 children with egg allergy	75
	66°C for 6 min	Pasteurized egg		=/↓ compared to raw egg		
	56°C for 6 min	Pasteurized EW		=/↓ compared to raw egg		

Protein fraction	Heating	Heating conditions	Product	Outcomes	Patient characteristics	References
Oral food challenges						
EW	90°C for 60 min	Liquid EW	Unheated or heated EW preparations (cum. dose 8 g), mixed in 50 mL of a thick liquid vehicle consisting of the fruit juices	21 of 38 subjects with positive challenge responses to freeze‐dried EW had negative challenge responses to heated EW.	72 participants with high IgE antibody titres to EW (a percent binding value that was higher than 3.1% in the RAST assay)	76
EW	90°C for 60 min	Liquid EW	Unheated or heated EW preparations (cum. dose 8 g) mixed in 50 mL of a thick liquid vehicle consisting of the fruit juices	29 children with a positive challenge to freeze‐dried egg had a negative challenge to heated EW. All heated EW responsive children were considered to be responsive also to freeze‐dried eggs.	108 children with suspected egg allergy	77
Whole egg	176°C for 30 min (muffin) or 260°C for 3 min (waffle)	Whole egg (1/3^rd^) + wheat matrix/muffin or waffle	The first muffin, then waffle. Each divided into 4 equal doses (total cum. dose = 2.2 g of protein)	Of 117 oral food challenges to BE, 87 patients were tolerant to BE and 27 reacted. Of the 87 tolerant patients, 39 reacted to a regular egg product in a follow‐up challenge.	117 patients with a clinical history of egg allergy between the ages of 0.5 and 25 years.	38
	Not specified	Not specified	Scrambled egg or French toast (total cum. dose 6.5 g of protein)			
Whole egg	180°C for 20 min	Whole egg + wheat matrix	Sponge cake or uncooked egg (pasteurized). Sponge cake: 5 incremental doses (0.4 g, 0.8 g, 1.5 g, 3 g, 6 g = cum. dose ap‐ proximately 1.0 g protein). Uncooked egg: 5 incremental doses (0.5 g, 1 g, 2 g, 6 g, 12 g = cum. dose approximately 2.6 g protein)	28/77 (37%) of well‐cooked egg and 61/104 (59%) of uncooked egg challenges were positive	95 children with a type‐1 hypersensitivity reaction to egg and/or SPT weal diameter ≥3 mm to whole egg extract, and/or serum egg‐white‐specific IgE ≥0.35 kU/L	78
EW	90°C for 10 min	Boiled	A starting dose of 0.12 g EW protein, with doses doubled consecutively (0.24–0.48–0.96–1.9 g) with hourly intervals until signs appeared (cum. dose 3.7 gEW protein)	50/85 children reacted to heated EW. Of the 35 non‐responsive children, 14 children reacted to unheated EW.	85 children aged 5–18 years on follow‐up for IgE‐mediated egg allergy, with positive sIgE(>0.35 kU/L) or SPT (>3 mm) to EW, OVA or OVM	79

Abbreviations: BE, baked egg; EW, egg white; OVA, ovalbumin; OVM, ovomucoid.

When the elicitation capacity was studied in a clinical setting, several studies showed that the wheal diameter of patient skin‐prick tests (SPT) using baked egg in the presence (*muffin*) or absence (*oven‐baked*) of wheat was generally smaller when compared to raw EW^73^, ^74^ (see Table 4). Similarly, using hard‐boiled egg, EW or egg yolk, part of children responsive to raw egg forms were not responsive any more in SPT (43% (egg), 33% (EW) or 72% (egg yolk)).^75^ Pasteurization of egg or EW did not significantly affect SPT size, and only very few raw egg reactive patients became non‐reactive upon pasteurization^75^ (see Table 4).

In oral food challenges (OFC) that investigate the clinical reactivity profile of egg‐allergic patients, a direct comparison of the reactivity towards baked/heated and uncooked eggs is generally not made (see Table 4). Instead, a patient who reacts to baked or heated egg is considered to react also to raw egg.^38^, ^76^, ^77^, ^78^, ^79^ These studies indeed show that a significant percentage of patients with a negative challenge to baked egg react to raw egg or a regular egg product (e.g. scrambled egg)^38^, ^76^, ^77^, ^78^, ^79^ (see Table 4). Given the more complex composition of the foods tested in SPT, the relative impact of heating versus glycation is difficult to be determined in these studies. Taken together, the heating or baking of EW (proteins) significantly lowers the capacity to provoke an allergic reaction, with the most pronounced changes observed after prolonged heating. Given the heat stability of OVM, it is likely that part of the residual immunoreactivity of heated EW is due to the recognition of OVM and not OVA. In support for a role of OVM in reactions towards heated egg, a part of patients responsive to heated egg was able to consume heated eggs depleted of OVM.^76^

**Summary:**

1

- OVA heating lowers its sensitization capacity, while the impact of OVM heating or OVA/OVM glycation on sensitization capacity remains to be further investigated
- Heating of EW (proteins) lowers its capacity to induce an allergic reaction in mice
- Heated or baked egg white (proteins) has a lower sIgE binding capacity and lower SPT wheal diameter compared to raw egg
- A significant proportion of egg‐allergic patients irresponsive to heated/baked egg (white) react to raw egg in OFC
- The impact of heating or baking on allergic reactivity is dependent on the time and temperature of heating

### 2.3 Patient reactivity to heated/baked EW and patient prognostics depend on the sIgE sensitization profile

The previous sections highlight that heating has a significant impact on the sensitization capacity and allergic reactivity of EW by altering EW structure and digestion. However, to understand why certain patients react to heated egg whereas others do not, we also need to look at the patients' clinical profiles. Several studies have attempted to address this question. One recurrent and confirmed observation is that patients reactive to both heated and raw eggs are characterized by higher overall sIgE levels of EW, OVA and OVM and by larger wheal sizes following SPTs compared to patients responsive only to raw egg.^38^, ^40^, ^80^, ^81^, ^82^, ^83^ Similarly, reactivity threshold doses for children allergic to raw but not baked egg are higher than for the general population of egg allergic children.^84^ These observations suggest that the severity of egg allergy might be a determinant factor for being tolerant or reactive to heated eggs. However, although elevated sIgE has a predictive value for the classification of patients, no generalizable cut‐offs for SPTs or sIgEs have been agreed upon so far and an OFC using heated or baked egg remains the gold standard.^82^, ^83^, ^85^, ^86^

Given the heat stability of OVM, several studies have suggested that the sIgE levels of OVM might be used to discriminate patients responsive or tolerant to heated egg.^40^, ^77^, ^80^, ^87^, ^88^ However, other studies have not confirmed a predictive value of OVM sIgE levels and no cut‐off for patient classification on the basis of OVM sIgE is currently available.^82^, ^86^ One factor that might explain the discrepancy between studies is the usage of heated egg versus baked egg due to the aggregation of heated OVM in the presence of wheat.^51^, ^82^ However, to what extent the presence of wheat influences clinical reactivity to OVM in patients remains to be further established. A study that orally challenged egg‐sensitized individuals with different food matrices suggested that the presence of wheat was only important in a minority of the patients and that the duration of egg heating (10 min vs. 30 min) was more determinant for a clinical reaction.^89^ Beyond the magnitude of egg sIgE levels or OVM sIgE levels, a higher reactivity to linear epitopes (that are less heat‐altered) in patients reactive to heated egg might also play a role.^33^ This type of information is, however, not obtained by measurement of sIgE binding to the entire allergen and specific epitopes that might predict the tolerance or not to heated egg would need to be confirmed.^28^

#### 2.3.1 Patient prognostics

Beyond contributing to the quality of life of egg‐allergic patients, a patient classification based on responsiveness to heated eggs might be useful to anticipate patient prognostics. As mentioned, many patients will outgrow hen's egg allergy, with a resolution of approximately 50% at the age of 2.^5^ The ability to tolerate baked egg is predictive of the transiency of egg allergy; patients unable to tolerate baked egg are five times less likely to develop tolerance.^5^ In line with the characteristics distinguishing baked egg‐tolerant from reactive patients, it has been proposed that patients who have higher sIgE to raw EW, that are sensitized to OVM or multiple egg allergens and that are highly reactive to linear epitopes of OVM or OVA are less likely to outgrow their egg allergy.^33^, ^86^, ^90^, ^91^

**Summary:**

1

- Patients reactive to both heated and raw eggs are characterized by higher overall sIgE levels to EW, OVA and OVM compared to patients responsive only to raw egg
- Tolerance to baked egg is predictive of the transiency of egg allergy; patients unable to tolerate baked egg are five times less likely to develop tolerance

## 3 USE OF HEATED EGG FOR PRIMARY PREVENTION AND TREATMENT OF EGG ALLERGY

Given the prevalence of egg allergy, a large number of studies have investigated primary prevention or treatment strategies. These studies are different, both in their protocols and in their results, but also notably in their usage of different forms of eggs to achieve tolerance; for example, raw or heated egg (white). The different structure and immunological reactivity of the different forms of eggs make it of interest to assess whether and to what extent the primary prevention and treatment of egg allergy is impacted by the form in which the egg allergen is provided.

### 3.1 Use of heated egg for primary prevention of egg allergy

One well‐studied approach to prevent egg allergy in infants is the early introduction of egg proteins during early food diversification (at 4–8 months of life).^92^, ^93^, ^94^, ^95^, ^96^, ^97^ Several randomized controlled trials have been conducted to evaluate the efficacy of an early introduction of egg in infants to prevent egg allergy using different types and doses of egg proteins, and different patient populations (general population, high risk) (see Table 5). In these studies, the most commonly used form of egg was pasteurized raw egg (white) powder, which has equivalent allergenic properties compared to raw egg^98^ (see Table 5). Other studies used heated egg powder or boiled egg (see Table 5). A systematic review and meta‐analysis^99^ assessed the combined effect of the early introduction versus no early introduction of egg protein and the risk of developing an egg allergy in these randomized controlled trials. It concluded to an overall significant protective effect of early introduction of egg protein with a decreased relative risk of developing an egg allergy in the egg group versus control group of 0.60 (CI: 0.44–0.82). It is, however, important to note that a significant number of adverse reactions (31%,^94^ 6.1%,^93^ 8.1%,^92^ 7.1%^96^) was described, notably in studies using pasteurized raw egg (white) powder. In contrast, the PETIT study, which used heated egg powder, did not describe any adverse events.^95^ The incidence of adverse reactions might also be impacted by the daily dose of egg protein given, which was high in the STAR study^94^ that described a high incidence of adverse reactions, and low in the PETIT study.^95^ Beyond the safety profile, the efficacy might also be impacted by the type or dose of egg used, but none of the studies directly compared the use of different types of eggs in primary prevention. Nonetheless, it is clear that heated egg—with its good safety profile—is able to successfully prevent the development of egg allergy,^95^, ^97^ whereas the studies using pasteurized egg (white) powder gave more heterogeneous results (see Table 5). In line with this, an observational study noted that exposure to cooked egg (defined as boiled, scrambled, fried, or poached) but not to baked egg (defined as egg‐containing cakes or biscuits or similar products) induced the development of oral tolerance: at 4–6 months of age, the first exposition to cooked eggs reduced the risk of egg allergy compared with the exposition to baked eggs (OR, 0.2 [95% CI, 0.06–0.71]).^100^ Based on these data, it might thus be hypothesized that for the effective prevention of egg allergy, the exposition of an infant to egg epitopes should be high enough to induce tolerance but also low enough to not sensitize or provoke an allergic reaction. This balance might be modified not only by the dose of egg used but also by the form of egg protein given. Indeed, as discussed in Section 2, the heating of EW protein modifies the accessibility of linear and conformational epitopes and increases its digestibility.

**TABLE 5:** Randomized controlled primary intervention trials for egg allergy.

Study name	Population	Form of egg	Dose	Primary outcome	Main result	Reference
STAR	86 infants with moderate‐to‐severe eczema	Pasteurized raw egg powder	0.9 g of egg protein per day	Egg allergy on oral challenge and positive SPT to egg	A non‐significant reduction of IgE mediated egg allergy in the egg group compared with the control group	94
EAT	1303 infants from general population	Cooked egg (together with 5 other types of allergens)	2 g/week	Food allergy following oral food challenge	Intention‐to‐treat analysis: a non‐significant reduction of egg allergy in the early introduction group.Per‐protocol analysis: a Significant reduction of egg allergy in the early introduction group.	97
STEP	820 infants with hereditary risk	Pasteurized raw whole egg powder	0.4 g of egg protein per day	Egg allergy on oral challenge and positive SPT to egg	A non‐significant reduction of IgE mediated egg allergy in the egg group compared with the control group	93
BEAT	319 infants with hereditary risk	Pasteurized raw whole egg powder	0.35 g of egg protein per day	Sensitization to white egg based on SPT	A reduction in the proportion of infants sensitized to EW in the egg group compared with the control group	92
HEAP	380 infants from general population	Pasteurized EW powder	2.5 g per week, 3 times a week (equivalent to 0.83 g of egg protein 3 times a week)	Sensitization to hen's egg, based on increased specific serum IgE levels	A non‐significant augmentation of egg sensitized infants in the egg group	96
PETIT	147 infants with eczema	Heated egg powder	50 mg of heated egg powder (equivalent to 25 mg of egg protein and 0.2 g of boiled egg), then 250 mg per day of egg powder	Egg allergy on oral food challenge	A significant reduction of egg allergies in the egg group compared with the control group	95

Abbreviation: EW, egg white.

The choice of the egg form to introduce into an infant's diet is of particular importance as a significant proportion of infants are already sensitized to eggs before food diversification.^94^, ^96^ How these infants are sensitized to egg is not fully clear, but their sensitization might have occurred *in utero* through the transfer of small doses of antigen in breast milk or through a defective skin barrier (for example due to the presence of egg protein in dust).^101^, ^102^ Recent studies have suggested that the exposition of infants to egg‐derived allergens and egg‐specific IgG in breast milk might contribute to the development of oral tolerance and a lower egg allergic risk in infants.^103^, ^104^ An on‐going randomized controlled trial now aims to determine whether a higher maternal egg and peanut consumption during pregnancy and lactation might prevent the development on infant egg and peanut allergy.^105^

**Summary:**

1

- Heated egg might be the best form to prevent the occurrence of egg allergy, given its efficacy and safety profile. For this reason, the S3 guideline Allergy Prevention now recommends “For prevention of hen’s egg allergy, well‐cooked (e.g., baked or hard‐boiled), but no “raw” eggs (…) should be introduced with the complementary food and given regularly.”.^106^

### 3.2 Use of heated egg for egg allergy treatment: OIT

OIT is a potential treatment for egg allergy, consisting of the progressive reintroduction of the allergy‐causing food. It includes an induction phase (IP) during which the ingested dose increases progressively to reach a target dose, and a maintenance phase (MP) during which the allergen is taken regularly. The IP often starts with an initial escalation phase with increasing doses of allergen given every 20–30 min during a day or two under clinical supervision to determine the starting dose for the IP. Patients undergoing an OIT can achieve desensitization and sometimes achieve maintained tolerance. Desensitization refers to the ability to ingest a food without reaction while continuing to take regular doses of that food, whereas maintained tolerance is the ability to tolerate a food after a period of food avoidance. The maintained tolerance is assessed by performing an oral food challenge (OFC) after discontinuing the ingestion of the allergen for a period of at least 4 weeks.

Many studies have investigated the effectiveness of OIT in egg allergy, including randomized controlled trials, uncontrolled trials, and observational studies. We will focus here on 15 randomized controlled trials (see Table 6). Although many of these studies included only a few patients, the data provided by these studies indicate that the efficacy of egg allergy OIT is generally very good, although mild‐to‐moderate adverse events are very frequent (see Table 6). This observation was confirmed by a meta‐analysis that included 10 randomized controlled trials and concluded to the efficacy of OIT compared with a control group: most children (82%) in the OIT group could ingest a partial serving of raw or undercooked egg (1–7.5 g) compared to 10% of control group children.^123^ It should be noted, however, that in the different studies the inclusion criteria, dosage, target dose, and the duration of the IP and MP are diverse (see Table 6). Especially dosing and frequency of exposition seem quite important for tolerance induction, as demonstrated in the SEICAP study that compared two protocols of OIT.^115^ In this study, one group increased their daily egg intake with 5% and their weekly intake with 30%, whereas a second group had only a 30% weekly up‐dosing; the first pattern was more effective than the second.^115^

**TABLE 6:** Randomized controlled OIT trials for egg allergy.

Ref.	Population	Control	Type of egg	Dose	Duration	Primary outcome	Efficacy of desensitization	Efficacy of maintained tolerance	Adverse events
Dried powdered egg, pasteurized raw egg (white), dehydrated egg									
107	45 egg or milk allergic children (including 11 egg exposed and 10 egg controls)	Egg avoidance diet	Lyophilised egg powder	*IP*: Starting dose of 0.006 mg of egg protein, then dose increased based on individual tolerance to reach 2800 mg of egg protein *MP*: Minimum daily maintenance of 1600 mg egg protein.	*IP*: 67 days at least *MP*: 21 months *Egg avoidance*: 2 months	N/A	N/A	OIT: 36% showed permanent tolerance, 12% were tolerant with regular intake 16% were partial responders and 36% didn't complete the treatment because of adverse events.*Control*: 35% were tolerant at the end of the study.	All OIT children had mild or moderate side‐effects.
108, 109	55 egg‐allergic children	Placebo, then OIT after 2 years	Raw EW powder	*IP*: Initial day escalation starts with 0.1 mg of powder to reach at least 3 mg, then daily ingestion of powder to reach 2 g of powder.*MP*: Up to 2 g of egg‐white powder per day.	*IP*: 10 months maximum *MP*: 2 months at least and 4 years or until sustained unresponsiveness is reached. *Avoidance diet*: 4–6 weeks before an OFC.	Induction of sustained unresponsiveness on OFC with 5 g or 10 g of EW powder.	Desensitization at 10 months (OFC, 5 g): *Control*: 0% *OIT*: 55% At 22 months: (OFC 10 g) *Control*: 0% *OIT*: 75%	4–6 weeks of avoidance diet if OFC at 22 was passed: 28% of the children in the OIT group had sustained responsiveness. Year 4: 50% of the OIT group demonstrated sustained unresponsiveness	Oral or pharyngeal adverse events during 22 first months: *Control*: 78% *OIT*: 20% No severe adverse events occurred. During years 3 and 4: Mild symptoms were present in 54.5% of patients still dosing.
110	72 egg‐allergic children	Egg avoidance diet	Powdered pasteurized egg	*IP*: Escalation day starts with 1 mg of powder, then weekly increase of the dosage until a dose of 10 g of egg powder.*MP*: Diet including eggs	*IP*: 10 weeks on average *MP*: 12 months	Development of tolerance	*Control*: 21.8% *OIT*: 92,5%	N/A	*OIT*: 52.5% had gastrointestinal symptoms, with mild (38.1%) to more severe (61.9%) reactions.
111	31 egg‐allergic children	Placebo	Pasteurized dehydrated EW	*IP*: Starting dose of 0.1 mg, weekly administration of increasing dose which are doubled every week to reach 4 g in 4 months.*MP*: 1 cooked or boiled egg 3 times a week.	*IP*: 4 months *MP*: 6 months *Egg‐ avoidance*: 3 months	Achieved desensitization on DBPCFC at 4 months.	Desensitization at 4 months: *Control*: 0% *OIT*: 94%	After 3 months of withdrawal: *Control*: 7.1% *OIT*: 31%	*Control*: No adverse effects *OIT*: 29.4% had adverse effects.
112	61 egg‐allergic children	Egg avoidance diet	*IP*: Dehydrated EW *MP*: Undercooked egg	*IP*: Escalation day starts with 0.08 mg of EW protein to reach 140 mg. Then, weekly increase from 0.02 to 2808 mg. *MP*: 1 undercooked egg every 48h	*IP and MP*: 3 months followed by egg avoidance of 1 month	Induction of sustained unresponsiveness (DBPCFC at 4 months with 2808 mg of egg protein).	*OIT*: 93% were desensitized (in a median of 32 days)	DBPCFC passed at 4 months: *Control*: 3% *OIT*: 37%	*OIT*: 70% of patients had an allergic reaction during desensitization or maintenance phase.
113	33 egg‐allergic children	Egg avoidance diet	*IP*: Dehydrated EW *MP*: Undercooked egg	*IP*: Starting with 0.03 mg of EW protein. Up‐dosing several times the same day after 1 h without symptoms. In case of adverse events, the previously tolerated dose is ingested as the first dose the following day. Target dose of 2808 mg. *MP*: Undercooked egg every 48h	*IP*: Median of 3 days (range, 1–14 days) *MP*: 5 months	Desensitization to egg after 5 months of MP (the ability to eat 1 undercooked egg without or mild adverse events).	Desensitization at 5 months: *Control*: 0% *OIT*: 89.5%	N/A	*OIT*: Adverse events occurred in 69% of patients, mostly mild or moderate.
114	36 egg‐allergic children	Egg avoidance diet	Dried powdered egg	*IP*: 0.1 mg of powder increased every 3–4 days to reach a target dose of 4 g of powder. *MP*: 4 g of dried powder egg daily	*IP and MP*: 6 months	Percentage of patients able to tolerate 4 g of powdered egg without symptoms in the OFC at 6 months.	Children who passed OFC at 6 months *Control*: 0% *OIT*: 57%	N/A	*OIT*: 94.4% had allergic symptoms during treatment. 1 experienced anaphylaxis
115	101 egg‐allergic children	Egg avoidance diet	Powdered pasteurized EW	*IP*: Starting dose: 0.11 mg of egg protein Group PI: 30% weekly + 5% daily up‐dosing Group PII: only 30% weekly up‐dosing *MP*: 3.3 g of EW protein daily/every 2 days.	*IP*: 121.12 ± 91.43 days	Total desensitization at 12 months	*Control*: 16% *OIT*: 84,2% The PI pattern was more effective than the PII pattern.	N/A	89% patients developed adverse events: Mild (74,53%), moderate (21,9%) or requiring adrenaline (3.5%).
116	50 egg allergic children tolerant to BE	Egg allergic children untolerant to BE treated with OIT	*BE*: a Muffin or equivalent *Egg OIT*: Pasteurized raw EW powder	*BE*: 2 g of EW protein. *OIT*: *IP*: 0.1 mg up to 25 mg max of EW powder on escalation day, up‐dosing every 2 weeks. *MP*: Up to 2 g EW protein	*IP*: 10 months *MP*: 8 weeks at least the 1st year and during 2^nd^ year *Egg avoidance*: 8–10 weeks.	Development of sustained unresponsiveness	Year 1: *BE*: 7.4% *OIT‐ BE tolerant*: 56.6% Year 2: *BE*: 14.8% *OIT‐ BE tolerant*: 78.3%	*BE*: 11.1% *OIT‐ BE tolerant*: 43.5%	Similar in the BE group versus OIT‐BE tolerant group.
117	50 children with moderate to severe allergic reaction to egg.	Egg avoidance diet for 6 months, then OIT	*IP*: Pasteurized raw EW powder *MP*: Raw EW powder at least 3 times a week and boiled/fried egg/foods containing heated egg on the remaining days	*IP*: Starting dose: 0.1 mg of EW protein. The dose was increased weekly for the first 3 weeks and then biweekly *MP*: 1 g of EW protein	*IP*: 8 months *MP*: 3 months	The proportion of participants partially desensitized after 8 months of OIT (consumption of any dose below 1 g of EW protein without symptoms).	*Control*: 4.8% desensitized at 6 months. *OIT*: 8 months: 44% desensitized, 46% partially desensitized 18 months: 72% desensitized, 16% partially desensitized.	N/A	*IP*: 82% of the children experienced dosing symptoms, mainly mild to moderate gastrointestinal symptoms. No severe reactions were seen.
118	11 egg‐allergic children	Egg avoidance diet	Pasteurised liquid raw EW	*IP*: Starting with 0.1 mL of EW protein, with 12 increased levels to reach the target dose of 20 mL *MP*: 20 mL or 2.66 g of EW protein	*IP*: 5 days *MP*: 6 months	Tolerance of 40 mL of EW on OFC	*Control*: 0% *OIT*: 100%	N/A	N/A
BE									
119	43 egg‐allergic children tolerant to BE	Egg‐free baked products	BE (muffins, biscuits, cake).	10 g of BE (1.3 g of egg protein) 2–3 times per week. No dose increments.	6 months	Raw egg allergy on OFC 1 month after ceasing the intervention.	N/A	No significant differences in raw egg tolerance. *Control*: 33.3% *BE*: 23.5%	No significant differences in adverse events between groups: *Control*: 36.3% *BE*: 42.8%
Raw hen's egg emulsion or liquid raw EW									
120	20 children with severe egg allergy	Egg avoidance diet	Raw hen's egg emulsion	*IP*: Starting dose of 0.015 mL of undiluted emulsion. Doubling dose in hospital 5 times in 6 months, with increasing dose at home based on the frequency and severity of side effects, until reaching 40 mL.	6 months	Tolerance to between 10 and 40 mL of raw egg emulsion on OFC	*Control*: 90% reacted to the challenge (with dose <0.9 mL). *OIT*: 90% children achieved partial tolerance	N/A	All children in OIT group experienced adverse events.
121	20 children with moderate‐severe egg allergy	Egg avoidance diet	Raw hen's egg emulsion	*IP*: Starting dose of 0.27 mg of egg proteins. Dose doubled every 8 days until day 80, then doubled every 16 days to achieve a total daily intake of 25 mL of raw egg in 6 months.*MP*: Raw egg or food containing about 3 eggs 3 times/week	6 months	Daily intake of 25 mL of raw hen's egg emulsion	*Control*: 20% *OIT*: 80%	N/A	*OIT*: 50% presented symptoms
Low allergenic hydrolysed egg									
122	29 egg‐allergic children	Placebo	Low allergenic hydrolysed egg	9 g administered daily. No dose increments.	6 months	Percentage of children with a positive OFC	No significative difference was observed (36% in intervention group and 21% in controls).	N/A	*Control*: 14% of adverse events. *Treatment*: 46.6% of adverse events

Abbreviations: BE, baked egg; EW, egg white; IP, induction phase; MP, maintenance phase; N/A, not assessed or not reported; OFC, oral food challenge.

Different types of eggs were used in the different OIT trials (see Table 6). In general, most studies used a rather ‘native’ form of egg (white) for OIT trials, such as dehydrated egg, pasteurized egg (white) powder or liquid, or raw hen's egg emulsion. Dehydrated egg powder was most commonly tested and generally compared to a control group having either a placebo or an egg avoidance diet. Although different protocols were used, in all of these studies OIT was associated with an increased percentage of desensitization and maintained tolerance compared with the control group (see Table 6). One study that did not show efficacy used a low‐allergenic hydrolysed form of egg, but this study also did not use dose increments.^122^ Two randomized controlled trials specifically assessed the efficacy of baked egg consumption to induce oral tolerance in egg‐allergic patients,^116^, ^119^ as did one non‐randomized clinical trial.^124^ Indeed, earlier studies suggested that the regular ingestion of baked egg in egg allergic children could accelerate the development of egg tolerance.^5^, ^125^ In a small, non‐randomized clinical trial, the incremental ingestion of baked egg (from 125 mg to 3.8 g of baked egg daily) was shown to induce progressive desensitization to baked egg and lightly cooked egg (cooking conditions not specified).^124^ Importantly, compared to other OITs, only very few adverse events were reported.^124^ In contrast, in a randomized clinical trial that included a control group of egg‐avoiding patients, the regular ingestion of the same dose of 10 g of baked egg (equivalent to 1.3 g egg protein) for 6 months did not increase the proportion of patients who were able to pass an OFC to raw egg 1 month after ceasing the intervention.^119^ No significant differences in adverse events were reported between the baked egg‐consuming group and the control group.^119^ This study did, however, not use dose increments and cannot be officially classified as an OIT trial. Similarly, another randomized clinical trial assessed the efficacy of regular baked egg consumption (equivalent to 2 g EW protein daily, no dose increments) and compared this protocol to an OIT using pasteurized EW powder in baked egg‐tolerant patients (up dosing to 2 g pasteurized EW protein).^116^ In this study, regular baked egg consumption was less effective to induce sustained unresponsiveness than the OIT approach with pasteurized EW powder, with an equivalent safety profile.^116^ No randomized clinical studies have directly compared an OIT using baked or heated eggs with an OIT using a raw or pasteurized form of egg, although a randomized non‐controlled study suggested that heated eggs can be effectively used in OIT.^126^ Given the lower allergenicity of heated or baked egg, it might be hypothesized that the usage of baked or heated egg might provide a more favourable safety profile, especially in the initial steps of OIT. In some countries, so‐called food ladders are now tools used to progressively reintroduce common foods containing eggs into the diet of egg‐allergic children and to induce tolerance. These food ladders consist of a step‐wise gradual introduction of increasingly allergenic forms of egg at home, starting from extensively heated to less heated eggs. These food ladders could be considered as a form of OIT, but they still lack standardization and a sound scientific underpinning of their efficacy.^127^, ^128^

**Summary:**

1

- OIT is an effective approach to promote desensitization and maintain tolerance in egg‐allergic patients
- Dehydrated egg powder is the most commonly tested form of egg in OIT
- The usage of heated or baked forms of egg might be an option for OIT, but more research is needed to confirm preliminary studies

## 4 CONCLUSION

**FIGURE 3:**
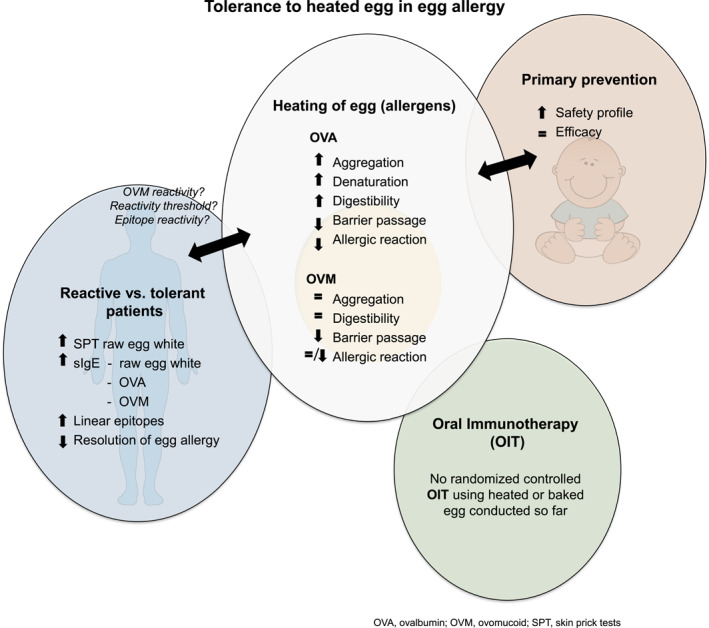
Overview of the physicochemical characteristics of egg white (EW) proteins and the patient characteristics that are potential determinants for the tolerance of patients towards heated eggs. The impact of egg heating on primary prevention strategies and oral immunotherapy is also noted.

To understand and establish strategies for the diagnostics, treatment and prevention of food allergy, detailed information about the responsible allergens is required. In the case of hen's egg allergy, a part of the patients is reactive to raw but not extensively heated or baked egg. The reasons for this seem to be multiple and relate to the physiochemical properties of the heated egg allergens on the one hand, and patient reactivity on the other hand (see Figure 3). Heating notably impacts the protein conformation and digestibility of the major EW protein OVA, whereas heating only impacts OVM upon prolonged heating or when wheat is present. On the patient side, the overall immunoreactivity towards hen's EW appears to be determinant for the discrimination of patient tolerant or reactive to heated or baked egg. Other implicated factors are patient reactivity to the heat‐stable OVM and to linear versus conformational epitopes, but these factors require further experimental validation. For primary prevention strategies of egg allergy, the use of a heated/baked form of egg might limit adverse reactions when compared to pasteurized raw egg powder and effectively prevent the egg allergy. A lightly heated or baked form of egg might also be an interesting option, in order to ensure that an individual is sufficiently exposed to egg epitopes to induce tolerance, but that the risk of sensitizing or provoke an allergic reaction is low. OIT seems to be a promising treatment for egg allergy, but significant adverse events have been reported. The use of heated or baked egg could be an interesting option to limit these adverse events, but the current literature is insufficient to conclude the efficacity of such an approach. Taken together, a good understanding of the impact of food transformation on its allergenicity might be helpful to ameliorate primary prevention and treatment strategies for food allergies.

## AUTHOR CONTRIBUTIONS

**Audrey Leau**: Conceptualization (supporting); formal analysis (equal); investigation (equal); writing—original draft (supporting). **Sandra Denery‐Papini**: Conceptualization (supporting); supervision (supporting); validation (supporting); writing—review and editing (supporting). **Marie Bodinier**: Conceptualization (supporting); supervision (supporting); validation (supporting); writing—review and editing (supporting). **Wieneke Dijk**: Conceptualization (lead); formal analysis (equal); funding acquisition (lead); investigation (equal); resources (lead); supervision (lead); visualization (lead); writing—original draft (lead).

## CONFLICT OF INTEREST STATEMENT

All authors declare that they have no conflicts of interest.

## Data Availability

Data sharing is not applicable to this article as no new data were created or analysed in this study.
